# “Ignite & engage:” A mixed methods evaluation of a peer-led, school-based substance use prevention program

**DOI:** 10.1186/s13722-025-00595-6

**Published:** 2025-08-11

**Authors:** Kenneth W. Verbos II, Arjola Agolli, Stephen Sandelich, Anthony Alvarado, Alma Jusufagic, Kenneth D. Croes, Aleksandra E. Zgierska

**Affiliations:** 1 Family Medicine Residency, McLaren Northern Michigan Rural Training Program, Petoskey, MI USA; 2 Department of Family Medicine, College of Human Medicine, Michigan State University, East Lansing, MI USA; 3 Department of Family and Community Medicine, Penn State College of Medicine, Hershey, PA USA; 4 Department of Emergency Medicine, Penn State College of Medicine, 500 University Drive, Hershey, PA 17033 USA; 5 Rise Together, Appleton, WI USA; 6 MedStar Washington Hospital Center and MedStar Georgetown University Hospital, Washington, DC USA; 7 University of Wisconsin Survey Center, University of Wisconsin-Madison, Madison, WI USA; 8 Departments of Public Health Sciences, and Anesthesiology and Perioperative Medicine, Penn State College of Medicine, Hershey, PA USA

**Keywords:** Prevention, Adolescent, Substance use, Addiction, Recovery

## Abstract

**Background:**

Substance use often begins in adolescence, making early identification and prevention essential to avert downstream harms, including substance use disorders. This study evaluated the impact of a peer-led, school-based storytelling program on participating middle- and high school students.

**Methods:**

A cross-sectional, anonymous, online survey was distributed from 2014 to 2020 to Midwestern middle and high-schoolers who had attended a school-based event, “Ignite & Engage,” led by a recovery community organization (RCO). Descriptive statistics summarized quantitative and an inductive thematic analysis summarized qualitative survey results.

**Results:**

Of 10,438 surveyed students, 2,853 (27.3%) reported ever using substances The majority (55.2%) reported initiating substance use between the ages of 14 and 16 years, and 29.2% initiated it at an earlier age. The program impact was rated by 996 respondents, with 71.3% of them viewing it favorably and 76.3% noting presenters’ personal recovery stories as the most valuable elements, and 51.2% feeling less likely to use alcohol or drugs afterwards, an effect stronger in middle- than high school youth (60.3% vs. 49.5%, respectively, *p* < 0.001). Qualitative feedback (*N* = 2,884) echoed the quantitative findings, emphasizing students’ greater intent to avoid substance use, seek help, support peers, and reflect on recovery.

**Conclusion:**

This school-based storytelling program, delivered by presenters with lived experience of addiction recovery, represents a promising, novel approach to substance use prevention and harm reduction among middle- and high school students.

## Background

Adolescence is a critical developmental period marked by rapid biological, mental, emotional, and social changes [1]. These transitions are often accompanied by increased susceptibility to risky behaviors, including substance use [2–4]. Research indicates that substance use during adolescence has profound short- and long-term consequences, including the development of substance use disorders (SUD), as well as psychological, social, and societal harms [5–7]. 

Alcohol, marijuana, and tobacco remain the substances most commonly used by adolescents, often beginning during early high school years [8–10]. Early initiation of substance use is particularly concerning, as it increases the risk of adverse outcomes such as addiction, criminal behavior, and mental issues [10, 11]. Recent data from the 2023 Monitoring the Future (MTF) survey indicates that approximately 62% of 12th graders, 76% of 10th graders, and 87% of 8th graders reported abstaining from alcohol, marijuana, cigarettes, and e-cigarettes in the past 30 days, marking record levels of non-use among adolescents [12]. Despite decreasing overall rates of adolescent substance use, the age of initiation among those who use substances appears to be decreasing, highlighting the urgent need for effective prevention strategies [5]. 

School-based programs are uniquely positioned to address substance use during adolescence, given their broad reach and cost-effectiveness [13–16], though their effectiveness is variable [17]. Evidence suggests that interactive and peer-led interventions—delivered by individuals with lived experience—may provide a promising alternative to traditional methods [13]. Rise Together, a peer-led recovery community organization (RCO), implemented the “*Ignite & Engage School Speaking Program*” to educate and empower middle and high schools about substance use, addiction, and mental health. Sharing personal stories, these events aim to reduce stigma and foster open conversation about these topics. This study leverages survey data collected from students who participated in the program to evaluate the scope of adolescent substance use and the impact of a peer-led, school-based intervention.

This study aimed to fill critical knowledge gaps about the perceived impact of peer-led prevention programs and their potential to serve as effective future school-based interventions for adolescent substance use-related harms.

## Methods

This manuscript follows the Consensus-Based Checklist for Reporting of Survey Studies reporting guidelines [18]. 

### Study design

This cross-sectional survey was conducted as part of a program evaluation by an RCO. The RCO’s school-based programming focused on the prevention and harm reduction related to substance use and mental health problems among middle and high-schoolers in the Midwestern United States. After attending an RCO-led event, students were invited to participate in an online, anonymous survey designed to assess their understanding of and personal history with substance use and mental health, as well as the impact of the RCO’s intervention.

The survey was distributed by the school, fully anonymously, with no identifying information collected by the RCO. These project evaluation efforts did not meet criteria for human subject research. As the Rise Together founder and member, one of the authors of this manuscript (AA) delivered most of the RCO’s programming sessions in schools; the remaining authors were not involved in the school events, or survey design or administration.

### Population

The survey was administered to students who attended at least one RCO-led school event between 2014 and 2020. These events were held at 240 middle and high schools across Wisconsin and adjacent states. Students were categorized as middle-schoolers if they were in grades 6–8 and high schoolers if they were in grade 9–12.

### School-based program

The RCO’s “*Ignite & Engage School Speaking Program*” aimed to educate and empower students to engage in discussions about substance use, addiction, recovery, mental health, and bullying. The presentation utilized storytelling by individuals with lived experience of addiction recovery to create a safe space for dialogue, reduce stigma, and encourage students to share their stories and seek help if needed.

Rise Together’s school-based programming is typically delivered as a single-day event, featuring two 60-minute school-wide or grade-level assemblies for middle or high school students. The assemblies are led by trained facilitators with lived experience of addiction recovery and focus on peer-driven storytelling about mental health, substance use prevention, addiction recovery, and personal empowerment. While the core sessions do not include structured group dialogue, post-presentation engagement often includes informal conversations between facilitators and students, classroom-based writing prompts, reflection activities, and group discussions. Schools selecting enhanced programming may receive follow-up resources (e.g., after-school club visits, optional storytelling workshops). Rise Together offers peer leadership initiatives and adult professional development activities for sustained impact to create long-term school culture change.

### Survey design and administration

The survey included questions on demographics, substance use, SUDs, mental health, recovery, community needs, and feedback on the RCO’s program. Questions evolved over six years, ranging from 40 to 48 questions (median: 45). The survey comprised multiple-choice and open-ended questions for qualitative insights; respondents could skip questions. Over this timeframe, the wording of some questions and response options changed slightly, and some questions were dropped.

Surveys were administered via the SurveyMonkey^®^ platform. School administrators distributed survey links to students after each event; participation was voluntary and uncompensated. Only students who answered “Yes” to the question, “*Have you ever used alcohol or other substances?*” were included in the quantitative analysis of substance use-related issues.

The perceived impact of the event was assessed through three quantitative questions: (1) “*Did you like the Rise Together Presentation?*” (Yes/No); (2) “*What did you like about the Presentation?*” (response options: Information about drug use, Presenters are relatable, Hearing the personal stories, Learning how to make a difference in my own community, Being motivated, Learning about Rise Together, Nothing, Other); and (3) “*After seeing a* Rise Together *presentation*,* how likely are you now to use drugs and alcohol?*” (response options: Much Less Likely than Before, Less Likely than Before, Same as Before, More Likely than Before, Much More Likely than Before).

An additional open-ended question allowed for qualitative responses; this question evolved over time from its original 2014–2016 version (“*Would you like to provide a personal testimony to share with us?*”) to its 2016–2020 version (“*Show us some love! Tell us what type of positive impact Rise Together made on you by leaving us a comment here*”).

The survey also assessed substance use history, using a “Yes/No” response format for alcohol, tobacco, marijuana, ecstasy, prescription drugs, heroin, cocaine, methamphetamines, and “other substances.” Students were asked about the presence of an impact of substance use, with “Yes/No” responses: substance use-related problems at school/work/home; affected relationships; abandonment of previously enjoyed activities; continued use despite the known risk; or withdrawal symptoms. The survey further inquired (“Yes/No”) about identifying themselves as being in recovery; help-seeking; prior assistance for substance use; and history of professional help (“Yes/No” to inpatient/residential treatment longer versus shorter than 30 days; outpatient treatment; detoxification; management of withdrawal); and support group attendance (e.g., Narcotics Anonymous or Alcoholics Anonymous).

### Analytical approach

Quantitative data were managed using STATA Version 11.0. Descriptive statistics, including frequencies, mean ± standard deviation, and SD, were described as quantitative data using the IBM SPSS software (Version 28.0). Qualitative responses were analyzed using inductive thematic coding [19]. Qualitative responses were coded by two analysts, led by one of the authors (KC), from the University of Wisconsin Survey Center, using the NVivo software (Version 12). The coding team developed the codes inductively from the responses themselves; they did not use an a priori coding scheme. The coding team used the initial coding method19 and grouped initial codes into themes as coding progressed. Both analysts participated in the coding of all responses in the following way. One analyst served as the initial-coder. The other analyst reviewed the initial coding. The reviewer-coder was free to disagree with the initial coding and create and apply different codes than the initial coder. In all cases of coding disagreement, the initial-coder and reviewer-coder discussed their differences until consensus was reached. Code definitions were refined as needed to improve agreement.

## Results

### Baseline characteristics

A total of 10,438 students completed the survey (Table 1). The majority identified as female (54.7%). Approximately 36.1% of the sample were in middle school (grades 6–8), and 63.1% were in high school (grades 9+). The largest age group was represented by 10–14-year-old (45.4%), followed by 15-16-year-old (33.3%) and 17–18 years (20.4%) students. Among the survey respondents, 27.5% (*N* = 2,853) responded “Yes” to the question: “Have you ever used alcohol or other substances?”.

**Table 1:** Demographics and age of substance use initiation among students who completed the survey (*n* = 10,438)

Variable	Respondents (*n* = 10,438)
**Gender, # (%)**	
Female Male Transgender Nonbinary Other	5,707 (54.7) 4,250 (43.3) 94 (0.9) 41 (0.4) 76 (0.7)
**Race, # (%)**	
White Black or African-American Asian American Indian or Alaskan Native Native Hawaiian or Other Pacific Islander Multiple races Prefer not to answer	8,997 (86.2) 187 (1.8) 226 (2.2) 128 (1.2) 38 (0.4) 471 (4.5) 391 (3.7)
**Grade, # (%)**	
**Middle school** 6th grade 7th grade 8th grade **High school** 9th grade 10th grade 11th grade 12th grade	**3**,**766 (36.1)** 1,102 (10.6) 1,449 (13.9) 1,215 (11.6) **6**,**586 (36.1)** 1,941 (18.6) 1,768 (16.9) 1,524 (14.6) 1,353 (13.0)
**Age, # (%)**	
10–14 years old 15–16 years old 17–18 years old > 18 years old	4,743 (45.4) 3,476 (33.3) 2,126 (20.4) 93 (0.9)
**Have you ever used alcohol or other substances?, # (%)**	
No Yes Missing response	7,526 (72.1) 2, 853 (27.3) 59 (0.06)
**Age of substance use initiation, # (%) (** ***N*** ** = 2,853)**	
≤ 8 years old 9 years old 10 years old 11 years old 12 years old 13 years old 14 years old 15 years old 16 years old 17 years old 18 years old > 18 years old	111 (4.5) 41 (1.7) 90 (3.6) 83 (3.3) 214 (7.5) 338 (8.6) 446 (18.0) 512 (20.7) 408 (16.5) 188 (7.6) 39 (1.6) 9 (0.4)

Among 2,853 students who reported lifetime substance use (Table 2), 55.1% identified as female and 86.0% as White, with 86.9% attending high school. They reported mean age of substance use initiation as 13.9 ± 2.3 years, with 55.2% initiating it at age 14–16 years, 9.8% at age 10 years or younger, and 4.5% at age 8 years or younger. Alcohol, marijuana, and tobacco were the most frequently reported substances used. In the group of students reporting a history of substance use, 56.9% (*N* = 1,624) responded to questions regarding substance use-related problems, with 10–15% of them reported negative consequences, such as relationship, academic, or health problems., without significant differences between middle and high schoolers in the frequency of these consequences. In this sample, 2,639 students answered questions about asking for help, with 241 (9.1%) reporting having asked for help with their substance use in the past and 110 children (8.6%) reporting receiving help and being now in recovery, including 61 self-identifying as being in long-term recovery; there were no significant differences between middle and high schoolers in their responses. Among the 128 respondents (21 middle. 107 high schoolers) who reported on the types of help received, outpatient treatment (55.8%) and self-help group attendance (55.5%) were the most common. High schoolers were more likely to report prior outpatient and withdrawal treatments than middle schoolers (*p* < 0.05).

**Table 2:** Substance use experiences among middle and high school students who answered ‘yes’ to ever using alcohol or other substances (n = 2,853). For questions where responses were missing, the sample size for the number of respondents to a given question is presented

Question	Total sample (*n* = 2,853)	Middle schoolers (*n* = 375)	High schoolers (*n* = 2,478)	*p*-value^1^			
**Substance tried or experimented with, # (%)**							
Alcohol Marijuana Tobacco Prescription pills (not prescribed to me or not used as prescribed) Cocaine Ecstasy Methamphetamines Heroin Other	2,441 (85.6) 1,178 (41.3) 1,076 (37.7) 437 (15.3) 121 (4.2) 99 (3.5) 46 (1.6) 38 (1.3) 155 (5.4)	281 (74.9) 70 (18.7) 78 (20.8) 47 (12.5) 6 (1.6) 2 (0.5) 5 (1.3) 3 (0.8) 43 (11.4)	2,160 (87.2) 1,109 (44.8) 998 (40.3) 390 (15.7) 115 (4.6) 97 (3.9) 41 (1.7) 35 (1.4) 112 (4.5)	< 0.001 < 0.001 < 0.001 0.09 0.02 < 0.001 0.52 0.28 < 0.001			
**Negative consequences of substance use**,** # (%)**	***n*** ** = 1,624**	***n*** ** = 153**	***n*** ** = 1,471**				
***Substance use causes problems at home, work, or school***							
Yes No	151 (9.3) 1,473 (90.7)	17 (11.1) 136 (88.9)	134 (9.1) 1,337 (90.9)	0.23			
***Substance use causes problems in relationships***							
Yes No	237 (14.6) 1,387 (85.4)	18 (11.8) 135 (88.2)	219 (14.9) 1,252 (85.1)	0.18			
***Activities you once enjoyed were given up due to substance use***							
Yes No	157 (9.7) 1,467 (90.3)	13 (8.5) 140 (91.5)	144 (9.8) 1,327 (90.2)	0.45			
***Continued use even when it has put you in danger.***							
Yes No	225 (13.9) 1,399 (86.1)	18 (11.8) 135 (88.2)	207 (14.1) 1,264 (85.9)	0.31			
***Withdrawal symptoms (nausea, headaches, vomiting, shakes, irritability, etc.) when you do not use.***							
Yes No	172 (10.6) 1,452 (89.4)	15 (9.8) 138 (90.2)	157 (10.7) 1,314 (89.3)	0.62			
**Help with substance use**,** # (%)**							
***Asked for help with your substance use.***							
Yes No				***n =*** **2639** 241 (9.1) 2398 (90.5)	***n*** **= 356** 33 (9.2) 323 (90.7)	***n =*** **2283** 208 (9.1) 2,075 (90.9)	0.94
***Received help and entered long term recovery.***							
Yes No	***n*** **= 1,283** 110 (8.6) 1,173 (91.4)	***n*** **= 134** 16 (11.9) 118 (88.1)	***n*** **= 1,149** 94 (8.2) 1,055 (91.8)	0.18			
***Identified themselves as being in long-term recovery.***							
Yes No	***n =*** **962** 61 (6.3) 901 (93.7)	***n*** **= 174** 8 (4.6) 166 (95.4)	***n =*** **788** 53 (6.7) 735 (93.3)	0.32			
***Type of help received (among those who reported getting help), # (%)***							
Inpatient/residential treatment > 30 days Inpatient/residential treatment < 30 days Detoxification unit Medical assistance with withdrawal Outpatient treatment Self-help group NA or AA	***n*** **= 128** ^**2**^ 28 (21.9) 34 (26.6) 15 (11.7) 17 (13.3) 65 (50.8) 71 (55.5) 19 (14.8)	***n*** **= 21** ^**3**^ 7 (33.3) 7 (33.3) 2 (9.5) 1 (4.8) 6 (28.6) 11 (52.4) 4 (19.0)	***n*** **= 107** ^**4**^ 21 (19.6) 27 (25.2) 13 (12.1) 16 (15.0) 59 (55.1) 60 (56.1) 15 (14.0)	0.10 0.40 0.66 0.04 0.01 0.72 0.48			

AA: Alcoholics Anonymous; NA: Narcotics Anonymous

^1^ Chi-square test

^2^ Response ‘did not receive help’ was provided by 1,774 (62.2%) and response was missing for 1,079 (37.8%) of the total sample of 2,853 respondents

^3^ Response ‘did not receive help’ was provided by 194 (51.7%) and response was missing for 181 (48.3%) of the sample of 375 middle school respondents

^4^ Response ‘did not receive help’ was provided by 1,580 (63.8%) and response was missing for 898 (36.2%) of the sample of 2,478 high school respondents

### Impact of the presentation (Table 3)

**Table 3:** Perceptions of the recovery community organization’s presentation among middle (*n* = 375) and high (*n* = 2,478) school students who answered ‘yes’ to ever using alcohol or other substances (n = 2,853). The number of students who answered a given question is presented at the top of each category; percentages are calculated within each category

Question	Total sample	Middle schoolers	High schoolers	*p*-value^1^
**After seeing a presentation are you less likely to use alcohol and drugs, # (%)**	***n*** ** = 996**	***n*** ** = 161**	***n*** ** = 835**	
Much Less Likely than Before Less Likely than Before Same as Before More Likely than Before Much More Likely than Before	229 (23.0) 281 (28.2) 433 (43.5) 17 (1.7) 36 (3.9)	66 (41.0) 31 (19.3) 53 (32.9) 5 (3.1) 6 (3.7)	163 (19.5) 250 (30.0) 380 (45.5) 12 (1.4) 30 (3.6)	< 0.001 0.002 0.001 0.12 0.91
**Did you like the presentation?, # (%)**	***n*** ** = 1,029**	***n*** ** = 173**	***n*** ** = 856**	
Disliked it Very Much Disliked It Neutral Liked It Liked It Very Much	35 (3.4) 38 (3.7) 222 (21.6) 306 (29.7) 428 (41.6)	11 (6.3) 5 (2.9) 40 (23.1) 45 (26.0) 72 (41.6)	24 (2.8) 33 (3.9) 182 (21.3) 261 (30.5) 356 (41.6)	0.02 0.41 0.66 0.09 1.0
**What did you like about the presentation?, # (%)**	***n*** ** = 2,005**	***n*** ** = 252**	***n*** ** = 1,** **753**	
Hearing the personal stories Presenters being relatable Information about drug use Being motivated to not use drugs Learning about Rise Together Learning about recovery Learning how to make a difference in my community Nothing	1,530 (76.3) 1,157 (57.7) 899 (44.8) 871 (43.4) 822 (41.0) 743 (37.1) 603 (30.1) 172 (8.6)	166 (65.9) 126 (50.0) 135 (53.6) 122 (48.4) 121 (48.0) 101 (40.1) 98 (38.9) 31(11.9)	1,364 (77.8) 1,031 (58.8) 764 (43.6) 749 (42.7) 701 (40.0) 642 (36.6) 505 (28.8) 141 (8.0)	< 0.001 0.01 0.004 0.18 0.06 0.31 0.002 0.05

^1^ Chi-square test

The Rise Together presentation and its impact were rated by those who reported substance use (*N* = 2,853).

The question: “After seeing a Rise Together presentation, how likely are you now to use drugs and alcohol?” was answered by 996 of respondents, with 51.2% of them rating being “less likely” or “much less likely” to use drugs; middle-schoolers were more likely to report this change than high-schoolers (60.3% vs. 49.5%, *p* < 0.001).

When asked: “*Did you like the Rise Together Presentation?*,*”* 1,029 of 2,853 respondents, with 71.3% of them reported “liking” or “liking very much” the event; however, 6.3% of middle-schoolers reported they “disliked it very much” compared to 2.8% of high-schoolers (p = 0.02), without significant differences observed in other satisfaction categories.

When asked: “*What did you like about the Presentation?*”, 2005 of 2853 students responded, with 76.3% marking the personal stories shared, 57.7% valuing the relatability of presenters, and 44.8% appreciating the information on drug use.

High-schoolers were significantly more likely to endorse “Hearing personal stories” (*p* < 0.001) and “Presenters being relatable” (*p* = 0.01), while middle-schoolers were significantly more likely to highlight “Information about drug use” (*p* = 0.004) and “Learning how to make a difference” (*p* = 0.002); no significant differences between middle- and high-schoolers were found for “Motivation to not use drugs,” “Learning about RT,” or “Learning about recovery” aspects of the presentation.

### Qualitative findings (Table 4)

**Table 4:** Thematic analysis: main categories and their major themes with corresponding representative quotes from individual respondents (*n* = 2,884)

*Category 1: Comments on the Rise Together Program (n = 608)*
**Theme: General positive comments**,***n* = 255** • Female, 6th grade: *“It was very informational*,* touching*,* and a little sad. I really hope that you can stop others from doing drugs. Thank you.”* • Female, 11th grade: *“I really like Rise Together’s message and I hope that you guys k’ep doing what you’re doing because drugs in high schools are an issue despite the fact that people like to say that they are not. The only ones in my school*,* that I have spoken to*,* that are pro-drug use are those that regularly use them. I feel that there is no need to do drugs at this day and age*,* or ever for that matter. They are cutting off neural signals to their growing brains and causing under development*,* not to mention a slew of other bodily issues. My mother raised me on the idea that drugs are dangerous and addicting; that if you do it once*,* then you will want to do it again.”* **Theme: Liked the presenters’ stories**,***n* = 116** • Male, 6th grade: *“It was very good and my favorite part was when that first guy talked about his life story.”* • Female, 9th grade: *“The second I heard […] start speaking I knew this wasn’t going to be an ordinary presentation*,* which is wasn’t. It was so powerful to hear those very real stories. This is one presentation that I will never forget.”* **Theme: Presenters were relatable**,** authentic**,** personal**,** or brave**,***n* = 106** • Female, 8th grade: “They gave us real life experiences talking about what they went through.” • Male, 8th grade: *“i think you guys are brave standing up and saying all the things you’ve been through”*
***Category 2: Impact of the Presentation (n = 1,646)***
**Theme: It inspired**,** helped**,** motivated or impacted us in an overall positive way**,***n* = 151**
• Female, 7th grade: *“I was inspired by Rise Above because I know there are so many people who have gone through similar things as the people who shared their stories*,* but some people might not have enough confidence or strength to talk about their experiences. So the fact that Rise Above is able to do that is super cool.”* • Female, 10th grade: *“Hearing your story helped me A LOT. I used to smoke 2 cigs a day*,* I now am officially done for 4 weeks. I get major withdrawals*,* but I go to my parents and they help me through them. I have medical issues*,* so alcohol puts me in hospital so I do not use anymore*,* buy weed is a problem because I do not want my family knowing I use.”*
**Theme: It inspired us to avoid substances**,***n* = 161**
• Male, 8th grade: *“I was very moved by all those people who have the confidence to go in front of people and tell their story or their loved one story. It really opened my eyes to not do drugs and also not experiment with drugs and alcohol”* • Female, 7th grade: *“Rise Together showed me how tough it can be when you take drugs and when you recover. It can be a scary thing*,* and I will never do drugs or anything like it.”*
**Theme: It inspired us to help others or showed us how to help**,***n* = 142**
Female, 7th grade: *“It made me think of drugs a different way then I did before. Several members of my family were/are addicts. It helped me to talk to them about their addictions.”* Male, 6th grade: *“Rise together made it more comfortable for me to ask and talk through drugs*,* Suicide*,* Or self-harm. Thank you for coming to are school and telling us how to help.”*
**Theme: It inspired us to share stories or open up or talk**,***n* = 111**
• Female, 8th grade: *“Well I think all three of the people’s stories were amazing ad inspiring. I also really liked how you let kids talk with you. Recently my cousin committed suicide and was really hard on my whole family and was our only boy cousin and my brother took it really hard and it helped when he talked with you guys!”* • Female, 10th grade: “*After hearing the speakers stories*,* I finally realized that I wasn’t alone. I even opened up to one of the speakers about a traumatic experience that happened to me years ago*,* and how it’s still affecting me today. During the whole presentation*,* I cried because some of the things they said I left sorry for them and I wished there was a way I would have been able to help them. I even went up in the front of my school and talked about how I’ve been affected by bullying*,* and I wasn’t afraid to do it even though things like that normally scare the life out of me. I told one of the speakers about a couple people who have told me about how miserable their life is*,* and I explained how I helped them. It felt good to talk about it*,* and I loved that they gave me advice and listened to what I had to say. When they talked to me they talked to me like I was a human being and told me how it was*,* and they gave their honest opinion*,* I felt so safe when I talked to them. On top of that*,* I was having a terrible week*,* and their presentation helped so much and for the first time that week*,* I was able to walk away with a real smile on my face*,* and not the fake one I had been wearing all week”.*
**Theme: Learned about dangers or consequences of drugs**,***n* = 255**
• Female, 6th grade: *“I learned that drugs and alcohol are not something you want to get attached to. Getting addicted to these things is so risky you can die from an overdose or the drug just kills you”* • Female, 8th grade: *“I always knew like I would never do drugs at all or drink (addicted wise) and then after hearing Doug’s story I was like even more than 100% sure because that’s something I never want to go through or experience. People always say don’t start cause it’s so hard to stop but hearing about his story really showed me how true that is and makes me even more sure about distancing myself from those kind of things.”*
***Category 3: Reports of Substance Use (n = 270)***
**Theme: Experiences with substance use**,***n* = 270** • Female, 7th grade: *“My aunt died of a heroin over does [sic] and the person who gave her the drug only went to prison for three months. I think he should have gone for a lot longer. But after rise together I know now that just because my friends or family use it’ll mean i should use”.* • Other, 11th grade: *“My father has been dealing with addiction for a very long time. He comes from a home where both of his parents were addicts and neglected him*,* my mom often says that compared to his parents he is a saint but th’t doesn’t excuse what he is doing to my mom*,* to me*,* my sister*,* and most of all to himself. Two years ago my depression reached its height*,* I was hearing voices that were telling me to hurt myself*,* others and eventually to kill myself. For over a year he noticed nothing because he was too drunk of his ass to realize his child was preparing to commit suicide. It took seeing my mom and family after a year apart for me to realize it was not something I could handle alone and that i needed help. Eventually It got to the poi’t where I’m afraid to be around him when he is drinking*,* he has never hurt me but he gets so angry and the look on his face… He looks like he would like nothing more than to beat me with the bottle in his hand. Other times I come home and I can hear music from outside our apartment building. I open the door and the counter is littered with bottles*,* the floor is a mess and he is passed out. The fact that I need to check if my father is still breathing is unacceptable because I know in my heart that one day I’ll come home and I will find him dead. He won’t be there to walk me down the aisle*,* to meet his grandchildren or be proud of me when I become a marine. I love my father*,* he’s my dad. He is so loving and goofy when he’s sober*,* but I’m not sure if I can let myself love him anymore*,* not if he is going to do this to his family. I know he has the willpower to quit but he won’t. I don’t know what’s worse anymore; knowing and loving him*,* to just lose him when I need him the most or to never have known him and save my family the heartache”.*
***Category 4: Recovery Stories (n = 121)***
**Theme: Stories of recovery from addiction**,***n* = 88** • Female, 9th grade: *“When your group “Rise Together” came to my school and did your presentation*,* l sat near the front and listened to the whole thing closely*,* it was crazy how much I can relate to some of your stories in the younger years of your life. Completely honest*,* I have just recently started the road to recovery from prescription pills*,* pot and alcohol. It’s been a little over a week since you came here and the day you came*,* was the day I decided I could make a change for the better in my life and did. Thank you so much for coming and sharing your stories with us*,* I do believe it made a lot of people realize what they were doing*,* including myself for sure. You all have incredible strength and courage and you’re all amazing people. Again*,* thank you so much for coming”* • Female, 11th grade: *“I would overdose on my anti-depression pills*,* because I wasn’t felling happy so I dealt with it myself. It was a high dose and I was taking about 5 or 6 at a time. I did this for about a year*,* and then my parents had me tested to see what was wrong and found out that I have a severe depression disorder*,* along with ADHD. Now I have control of my life and I’m at a charter school*,* and will graduate early.”* **Theme: Stories of others’ addiction recovery**,***n* = 33** • Female, 8th grade: “*My dad used to heavily drink. He would come home every night drunk and he would scream at my mom. It got so bad that my mom left us. Twice. Then one night me and my brother had to sleep at my grandma’s house because my dad had not yet come home. At around midnight*,* my grandma got a call from Theda. My dad had gotten in an accident and had to be flown via Flight of Life. He broke his back*,* neck*,* and his face was pretty much unrecognizable. He hasn’t had a drop to drink since. Now he is Remarried and acts like the dad he wishes he would have been back then. He’s pretty much my role model.”* • Male, 7th grade: “*I was able to help one of my friends who was struggling. Since I have grown up being rigorously told and made aware of drugs*,* this didn’t make too much of an impact on me. But it did on my friend*,* so it’s a win in my book.”*
***Category 5: Reports of Self Harm (n = 18)***
**Theme: Reports of self-harm**,***n* = 18** • Female, 8th grade: “*My mom had done many drugs and drank while I was in her stomach. I grew up not really knowing who she was. She always had new boyfriends and did drugs and drank while I was around. I have seen so many things I can’t erase from my mind. I have tried some of the things she has done and i did not care. I cut*,* I have wanted to die. But lucky I am better with the help from a close family member. But still am not 100% but I will be someday”* • Female, 7th grade: *I used to cut when my father abused me.*
***Category 6: Various Viewpoints Regarding the Substance Use Epidemic (n = 101)***
**Theme: Improve treatment availability**,***n* = 38** • Female, 11th grade: “*I think a reason people turn to drugs is when they can’t deal with a situation and there no one there to turn to. If there was an online community or a free camp they could go to for help*,* it may help lead them down a safer path than drugs”* • Male, 6th grade: *“we need to have a place for kids/adults to feel open and to be able to get the help they need without depression”* **Theme: Educate on drugs or alcohol**,***n* = 27** • Female, 6th grade: “*We need to bring heroin up more in school health classes. We don’t ever talk about the long term effects or even just the effects it can do to you.”*
***Category 7: Questions***,*** Feedback***,*** and Request (n = 121)***
**Theme: Feedback and requests for help and Rise Together presentations**,***n* = 94** • Female, 6th grade: “*Please help by making sure that everyone is safe from this.”* • Female, 12th grade: *I loved how y’all talked to us like adults. You didn’t stand up and say “Okay kids*,* drugs are bad*,* X amount of people die*,* have a great day!“. It was also lovely how y’all stayed after to talk to us*,* individually. It made all of us feel loved. The only thing I have to say is*,* perhaps you could allow 15 min or so for us to come down*,* take the mic*,* and share? Seeing all those people stand*,* people I never would have pegged to have problems… It was impactful. Thank you so much for coming.* **Theme: Negative feedback on the Rise Together survey**,***n* = 19** Female, 12th grade: “*This survey generalizes students. There are not enough choices for students like myself who do not have chronic use issues and it becomes frustrating very fast.”* Male, 12th grade: “*The survey makes you answer questions not intended for some people”*

Among the entire sample of respondents (*N* = 10,438), 2,884 students provided a qualitative response about the programs impact. Thematic analysis of these responses led to identifying seven main categories of themes: (1) program comments (2), presentation impact (3), substance use experiences (4), recovery stories (5), self-harm reports (6), viewpoints on the substance use epidemic, and (7) questions/requests.

Students frequently expressed appreciation for the program. One student noted, *“It was very informational*,* touching*,* and a little sad. I really hope that you can stop others from doing drugs. Thank you.”* Another student reflected on the broader impact, stating, *“I really like Rise Together’s message and I hope that you guys keep doing what you’re doing because drugs in high-schools are an issue despite the fact that people like to say that they are not.”* The storytelling aspect of the presentation resonated strongly with many students. One student stated, *“It was very good and my favorite part was when that first guy talked about his life story.”* Another described their reaction, saying, *“The second I heard […] start speaking I knew this wasn’t going to be an ordinary presentation*,* which it wasn’t. It was so powerful to hear those very real stories. This is one presentation that I will never forget.”*

Many students found the presenters to be relatable and inspiring; one shared, *“They gave us real life experiences talking about what they went through*,*”* while another remarked, *“I think you guys are brave standing up and saying all the things you’ve been through.”* For some, the presentation provided motivation to change behaviors. One student stated, *“Hearing your story helped me A LOT. I used to smoke 2 cigs a day*,* I now am officially done for 4 weeks.”* Another shared, *“Rise Together showed me how tough it can be when you take drugs and when you recover. It can be a scary thing*,* and I will never do drugs or anything like it.”* Several respondents noted that the presentation empowered them to help others. One student wrote, *“It made me think of drugs a different way than I did before. Several members of my family were/are addicts. It helped me to talk to them about their addictions.”* Another reflected, *“Rise together made it more comfortable for me to ask and talk through drugs*,* suicide*,* or self-harm.”* Finally, the presentation prompted students to share their own experiences. A student disclosed, *“After hearing the speakers’ stories*,* I finally realized that I wasn’t alone. I even opened up to one of the speakers about a traumatic experience that happened to me years ago*,* and how it’s still affecting me today.”*

## Discussion

This evaluation of Rise Together’s program suggests that peer-led, storytelling-based interventions in schools can be a powerful tool for engaging adolescents, increasing awareness about substance use risks, reducing stigma, and encouraging help-seeking behaviors, particularly when delivered by individuals with lived experience.

Among the 10,438 surveyed students, 2,853 (27.3%) reported a history of substance use, with alcohol, marijuana, and tobacco being the most commonly reported substances used. The majority of students initiated substance use during early high school years, yet a concerning proportion reported use beginning in middle or even elementary school. These findings underscore the urgent need for targeted, early prevention strategies to mitigate long-term risks associated with substance use initiation during adolescence.

Our findings align with national trends reported by the 2023 MTF survey which found that 26.2% of 12th graders, 16.9% of 10th graders, and 9% of 8th graders reported past-month any drug use, whereas our study found a higher proportion of students endorsing lifetime substance use. Additionally, the prevalence of marijuana use in the past month was reported at 29.0% among 12th graders, 17.8% among 10th graders, and 8.3% among 8th graders, which is comparable to our findings on the high prevalence of marijuana use. These national data highlight ongoing concerns regarding adolescent substance use despite overall declines in prevalence.

The impact of peer-led interventions in substance use prevention and harm reduction remains an evolving area of research. Our study suggests that a school-based program led by individuals with lived experience of addiction and recovery may provide an engaging and impactful approach to addressing this issue among adolescents. Over half of the students who attended the Rise Together event reported feeling less likely to use substances following the presentation, with middle-schoolers reporting a greater impact than high-schoolers. This aligns with previous research indicating that interactive, real-world storytelling may be more effective than traditional didactic prevention models in engaging adolescents and fostering meaningful behavioral change [16]. Storytelling-based interventions have been found to reduce students’ readiness for substance use and increase awareness of substance use-related consequences.

Our results also align with meta-analytic findings on the effectiveness of school-based interventions in reducing adolescent alcohol use. Prior research suggests that universal school-based programs produce small but positive effects on reducing alcohol consumption, particularly when interactive elements were incorporated [17]. Similarly, evidence-based prevention programs emphasize the importance of addressing risk and protective factors at multiple levels, including school, community, and family [13]. Our study contributes to this body of research by demonstrating that peer-led, storytelling-based interventions may provide an additional tool to enhance existing prevention strategies.

Qualitative responses further corroborated the effectiveness of the intervention. Students frequently cited the relatability and authenticity of the presenters as key factors contributing to the presentation’s impact. Many students expressed appreciation for the openness of speakers sharing their personal struggles, describing the experience as “powerful” and “eye-opening.” Others noted the presentation encouraged them to rethink substance use, seek help, or support others who struggle with addiction and mental health issues. These findings highlight the value of integrating personal narratives into substance use education, as such approaches may help reduce stigma, foster empathy, and promote open dialogue.

Despite these promising findings, the study identified significant gaps in adolescent substance use prevention and treatment. Although nearly 10% of substance-using students reported seeking help, only a fraction had accessed formal treatment services. High school students were more likely to report receiving outpatient care, while middle school students were more likely to report receiving inpatient treatment, suggesting potential differences in intervention accessibility or severity of substance use between age groups, underscoring the need for expanded access to adolescent-specific substance use treatment and support services within school and community settings.

### Limitations

Several limitations should be acknowledged. Typically, survey studies rely on carefully designed research, implemented with the assistance of research staff; the survey in this study was designed and implemented as a program evaluation tool and was not validated. Although the lack of research-level rigor serves as a limitation, collection of data on a large population of students (over 10,000 in this study) using a pragmatic evaluation approach to the real-world program, with students filling out the survey of their own accord, and without any additional incentives, e.g., financial compensation, represent strengths. Additionally, students’ response to the program was only analyzed for adolescents who noted substance use, leading to a possible selection bias.

Self-reported data are subject to bias, e.g., social desirability, recall or selection; students who voluntarily chose to complete the survey may differ from those who did not. Because the survey question asked about the use of ‘alcohol or other substances’, some students may not have answered affirmatively even if they had used tobacco/nicotine products, thus potentially underestimating tobacco/nicotine use prevalence. In addition, because the link to anonymous survey was distributed to students by school personnel, it precluded our ability to estimate a response rate or precise prevalence estimates of substance use among these adolescents. The lack of a control group limits our ability to assess the extent to which observed changes in attitudes toward substance use were directly attributable to the Rise Together program. The survey also did not specifically inquire about opioid use; therefore, the opioid use-related data were not collected systematically, limiting our ability to position the survey results in the context of existing opioid crisis. The 2016–2020 “Show us some love!” prompt could have introduced positive-response bias.

## Conclusions

Our findings suggest that storytelling, school-based programs, led by individuals with lived experience, may be effective in increasing awareness and reducing stigma associated with substance use, and encouraging help-seeking among adolescents affected by addiction. By fostering conversations and providing relatable role models, peer-led interventions may serve as a valuable tool in preventing substance use initiation and mitigating its harms among adolescents. Future research should rigorously assess the impact of such programs, including their ability to be integrated with existing school-based prevention frameworks and effectively influence actual substance use behaviors. Expanding access to adolescent-specific substance use prevention, treatment and support services remains a critical priority in addressing the broader challenges of youth substance use and addiction, and their downstream sequalae.

## Data Availability

The datasets used and/or analyzed during the current study are available from the corresponding author upon reasonable request.

## Abbreviations

SUD: Substance Use Disorder
RCO: Recovery Community Organization
MTF: Monitoring the Future
